# Advertisement of unreceptivity – Perfume modifications of mason bee females (*Osmia bicornis* and *O*. *cornuta*) and a non-existing antiaphrodisiac

**DOI:** 10.1371/journal.pone.0215925

**Published:** 2019-05-06

**Authors:** Karsten Seidelmann, Daniel Rolke

**Affiliations:** Martin-Luther-University Halle-Wittenberg, Institute of Biology, Department Animal Physiology, Halle (Saale), Germany; University of Illinois at Urbana-Champaign, UNITED STATES

## Abstract

Females of many monandrous insect species announce their receptivity either by specialised sex-pheromones or by a signature mixture of cuticular hydrocarbons (CHCs). The trigger that shuts down the sex-pheromone release or initialises a change in CHC bouquet is thought to be either the mating per se or male pheromones transferred during copulation. Besides a conversion of female volatiles, the application of antiaphrodisiacs, male derived pheromones that render mated females unattractive to competitors, is another strategy to protect females from further sexual chasings. This simple pattern becomes more complicated in the monandrous mason bees *Osmia bicornis* (syn: *O*. *rufa*) and *O*. *cornuta* due to a post-copulation phase in their mating sequence. Males display a stereotypic behaviour right after the intromission that induces females’ unreceptivity. This post-copulatory display is predestined both to trigger a transition of the CHC profile and for the application of an antiaphrodisiac. However, the postulated antiaphrodisiac was not detectable even on freshly mated females. Moreover, the male’s post-copulatory display did not trigger a change in the CHC bouquet and neither did the insemination. Instead the CHC profile of freshly emerged females changes into the bouquet of nesting females simply by age as an ontogenetic process in both *Osmia* species. This autonomous change in the CHC profile coincides with an age-specific decrease of young female’s willingness to mate. How the resulting short period of female receptivity without back coupling by storage of sperm and the lack of an antiaphrodisiac fit into the behavioural ecology of the studied mason bee species is discussed.

## Introduction

Females of most solitary bees are assumed to be monandrous and mate immediately after eclosion from the maternal nest to fill their spermatheca. Once mated, they become unreceptive and unattractive to males [1,2]. The status of receptivity is announced by the cuticular hydrocarbon (CHC) profile [3,4,5], a typical signature mixture [6]. Confusingly, the attractive CHC bouquet of virgin females was sometimes named “sex pheromone” [7,8,9]. The key stimulus both for the induction of unreceptivity and for the transition of the CHC profile in females is thought to be the copulation and filling of the spermatheca [4,10]. In the Red mason bee (*Osmia bicornis*), however, this intuitive control mechanism becomes more complicated because the intromission is not responsible for the induction of the unreceptivity. Instead, monandry is induced by a post-copulatory display [11,12]. Males remain mounted on the female after ejaculating and perform rhythmic buzzes accompanied by stereotypic movements of antennae, hind legs and abdomen for about 10 minutes. This behaviour induces female’s unreceptivity to further matings regardless of whether an ejaculate and sperm has been transferred or not [11]. Therefore, in *O*. *bicornis* both the sperm transfer or the post-copulation could serve as trigger for the change in the CHC bouquet of mated females. A second possible control mechanism for triggering the CHC transition arises in *Osmia* females from the close temporal connection of eclosion from the cocoon (inclusive leaving of the maternal nest) and mating. As adult age and probability of insemination are likely to be correlated, it is important to discriminate mating status from ontogenetic factors [4]. This subtle distinction becomes particularly relevant because virgin *O*. *bicornis* female’s willingness to mate decreases simply by age and independent from an effective copulation [11]. However, it is not known whether this age-related loss of receptivity is accompanied by a change in the CHC bouquet, and if so, whether the CHC alterations are similar to regular mated *O*. *bicornis* females.

Altogether there are three potential internal signals in line for triggering changes in the CHC profile of young females in *O*. *bicornis*. Two possible signals, sperm and post-copulatory display, are related to mating and one to age-related physiological processes as reproductive maturation. In order to distinguish between the two possible stimuli due to mating, males were removed from an inseminated female immediately after ejaculation and transferred to a freshly emerged, virgin female to perform their post-copulatory display. By this manipulation, both the CHC-profiles of inseminated females that had not received a post-copulatory display and of virgin females that experienced this display by a male could be analysed. The impact of age on the CHC bouquet was analysed by tracing the CHC profiles of both regular mated and virgin females over the first days as free-living adult after emergence from the cocoon. The transferability of the results to other mason bee species was validated by extending the study to *O*. *cornuta*, a syntopic and widely synvoltin [13] mason bee species with similar biology to *O*. *bicornis*.

Another aspect of intraspecific signalling by odour in young *Osmia* females is the proposed antiaphrodisiac as (Z)-7-hexadecenoic acid ethyl ester (7-HAD-EE) in *O*. *bicornis* [3]. The pheromone is produced in the sternal gland of the male and is probably spread over the female during the post-copulatory display [3,11]. The pheromone is assumed to act as a temporary deterrent from further male harassment until the female has changed her olfactory bouquet [11]. In dual-choice experiments, virgin females impregnated with sternal gland extracts were less attractive than controls [14]. However, the efficiency of a volatile for indirect mate guarding depends crucially on its perceptibility during the timespan of female’s CHC transition. The adherence of the considered antiaphrodisiac was therefore monitored in mated females for the first days after copulation.

The CHC profile of young females is crucial for central aspects of reproductive live. First females have to mate to obtain the sperm for their whole reproductive live span. They announce their receptivity to searching males by an attractive CHC profile. Once mated, females search for a suitable nest site and start nesting. To escape the permanent harassment of males for undisturbed nest building and provisioning, females should announce their unreceptivity by a transition of the CHC profile. The aim of our study was to document the characteristic differences in the CHC profile of receptive and unreceptive females, to identify the trigger, and to monitor the time course of alterations in the CHC profile. Finally, we wanted to prove how the postulated antiaphrodisiac fits into the CHC transitions to protect a freshly mated female for a time from further sexual harassment.

## Material and methods

### Animals and manipulation of mating status

*O*. *bicornis* and *O*. *cornuta* bees (cocoons or nesting females) were collected from a regularly maintained artificial rearing (bee-keeping of solitary bees) operated by the Institute of Biology / Zoology in the Botanical Garden of the University Halle-Wittenberg (Germany, Saxony-Anhalt, 51° 29' 04'' N, 11° 56' 07'' E; see [15] for details). No individuals of autochthone populations were used. Moreover, both mason bee species are not on the Red List of Endangered Species in Germany.

About one week after the last females had emerged in the rearing, samples of nesting females were collected at the bee shelters and frozen individually in glass vials. These bees (about 8 to 10 days old) were referred to as “nesting” or “brood active” bees to indicate that they are mated and aged at natural conditions. Females of known age and mating status were produced in the laboratory by using cocoons kept in stock at 4°C after wintering. Prior to experiments, bees were sexed according to the hair colour of the clypeus (males: tuft of white hairs; females: black hairs) by gently opening the cocoons at the top. Males and females were incubated separately to guaranty virginity of females. *O*. *bicornis* female mating status was manipulated by transfer experiments in a flight cage (0.62 × 1.26 × 0.62 m^3^) lighted by neon lamps (2.100 lux at the bottom of the cage) at L:D = 12:12 h. The temperature was kept at 22–25°C. Bees were fed on a honey/sugar/water solution (1/1/1). After establishment of regular flight activity of about 30 *O*. *bicornis* males within the cage, virgin females were released one by one into the cage. Immediately after one male has grasped a female and settled on her back the pair was gently transferred to the cage floor. Males were removed from females immediately after intromission by stripping them off with a cuvette lid that hosted another, virgin female. Males continued to perform their post-copulatory courtship display on the unmated female [11]. Both inseminated and post-copulated females were removed from the cage and kept separately in small plastic boxes at room temperature with honey/sugar/water solution at libitum for 3 to 8 days to allow CHC transition.

### Collection of CHC samples

Females were freeze-killed and the CHCs washed off from heads individually in small glass vials (1.5 ml) with PTFE sealed screw-caps (Phenomenex) by adding 100 μl of pentane (AppliChem). Vials were gently shaken to rinse the whole head surface with solvent. Heads were removed after allowing CHCs to dissolve overnight in the fridge at -21°C to prevent aqueous liquids from leaking out. The timespan of about 24 hours was chosen to generate an extract in equilibrium without discriminating persistent compounds. Extracts were stored at -21°C until analysed. CHC profiling was restricted to head samples in order to refine the chemical analysis. This approach seemed reasonable because males rush to females they have spotted and grasp them out of flight to reach the courting and mating position on the female’s back. After having only short contact to the female’s body, the antennae of the males can pick up information on her receptivity only from volatiles and the surface of the female’s head. For a sample of females beside the heads also the torsi were washed separately. This was done first, to confirm that samples of head and body represent the individual CHC profile in the same manner, the torsi of several females were extracted separately as well. Second, the proposed antiaphrodisiac is supposed to be applied by the male on wings and/or abdomen of his mate [3,11]. The application of 7-HDA-EE was tested by probing wings and torsi (bodies without head) of inseminated females immediately after, one, and two days post copulation. The surfaces of the body parts were washed by adding 50 μl (wings) or 300 μl pentane (torso) in 1.5 ml glass vials at -21°C overnight.

### Chemical analysis

Volatile bouquets were analysed by GC/EI-MS (Saturn 2100T, Varian, EI, 40–400 m/z) coupled to a CP 3900 GC (Varian) equipped with a VF-5ms column (30 m × 0.25 mm × 0.25 μm; splitless injection @ 220°C, helium @ 1.0 ml/min, 1 min @ 100°C, 20°C/min to 200°C, 5°C/min to 300°C, 5 min @ 300°C). Compounds were identified by NIST-database search while retention times (Covats-indices), and mass spectra were validated using pure reference substances. The double bond position of unsaturated CHCs was located by methylthiolation reaction with dimethyl disulphide adducts (DMDS, [16]).

### Statistical analysis

There were no distinct indicator substances present either in virgin or older mated (or nesting) females. Therefore, CHCs were treated as bouquet and all compounds detectable in every individual were used (Table 1). Due to the compositional nature of data, no internal standard was used and data analysis was based on relative proportions. Quantification of substances for the monitoring of age related changes were based on characteristic ions on mass spectra. However, to enable comparisons with other bee species and FID-data, area-percent of peaks were calculated on the basis of the raw ion counts (RIC). To handle a possible non-linear intensity bias due to e.g. body size depending total substance amounts, data were quantile-normalised [17] by the package ‘preprocessCore’ [18] for R [19]. Due to the compositional nature of the data, each normalised peak area was centred log-ratio transformed [20]: ${clr(Y}_{ij})=ln\left(\frac{Y_{ij}}{g(Y_{j})}\right)$ with Y_ij_ is the area of the peak i for bee j and g(Y_j_) is the geometric mean of all peak areas for bee j. The multitude of compounds was reduced by principal component analysis (PCA, regression method) extracting all factors with an eigenvalue > 1. A discriminant function analyses (DA) using all PCA factors was conducted on the PCA scores to distinguish freshly emerged (virgin) versus nesting (mated) females. The obtained discriminant function was used to calculate the discriminant scores for all females sampled as a measure of CHC transition and analysed by GLM (Wald test statistics) to identify an impact of age and mating. The conformance of head and torso extracts of one and the same individual were analysed for each 10 mated females one and two days old in *O*. *bicornis* and 8 freshly emerged (virgin) and 13 nesting (mated) females in *O*. *cornuta*, respectively. To handle potential problems of collinearity, the dimensionality of the data set was again reduced by PCA. Aggregation of samples in dependence of age and body part was determined by DA. Finally, a hierarchical cluster analyses was employed to calculate a similarity matrix based on squared Euclidian distances (SED). A higher similarity of head and torso of the same individual vs. head to torso of a different individual was tested by the difference of mean SED within an age cohort. Median and range were given in square brackets for samples with inhomogeneous variance. Error level was set to α = 0.05 and SPSS (IBM, V. 24) was deployed for statistical analyses.

**Table 1:** Area percentage composition (± SE) of compounds of head volatile extracts from *O*. *bicornis* (N = 55 freshly emerged, virgin; N = 12 nesting, mated), *O*. *cornuta* (N = 18 freshly emerged, virgin; N = 13 nesting, mated).

Peak	Compound	Retention Time	*O*. *bicornis*			*O*. *cornuta*		
			DA†	Virgin 0d	Nesting	DA†	Virgin 0d	Nesting
				% ± SE^‡^	% ± SE^‡^		% ± SE^‡^	% ± SE^‡^
1	7-C21:1	12.08 min		0.00	0.00	X	0.01 ± 0.01	0.02 ± 0.01
2	C21:0	12.36 min	X	0.77 ± 0.04	0.07 ± 0.01	X	1.02 ± 0.07	0.11 ± 0.02
3	9-C18:1-OOH	13.07 min		0.18 ± 0.07	< 0.01		T	T
4	5-C22:1	13.70 min	X	0.36 ± 0.02	0.45 ± 0.04	X	0.42 ± 0.05	0.28 ± 0.04
5	C22:0	13.75 min		T	T	X	0.21 ± 0.02	0.14 ± 0.04
6	X-C16:1-E^#^	14.14 min		< 0.01	< 0.01		0.18 ± 0.04	< 0.01
7	C16:0-E^#^	14.42 min		< 0.01	< 0.01		0.10 ± 0.02	< 0.01
8	9-C23:1	14.87 min	X	0.06 ± 0.01	0.08 ± 0.02	X	0.16 ± 0.02	0.71 ± 0.05
9	7-C23:1	14.98 min	X	0.07 ± 0.01	0.06 ± 0.01	X	0.72 ± 0.10	8.86 ± 0.69
10	5-C23:1	15.05 min		T	T	X	< 0.01	0.31 ± 0.04
11	C23:0	15.24 min	X	7.04 ± 0.23	0.85 ± 0.09	X	13.87 ± 0.49	6.04 ± 0.37
12	11-C24:1	16.29 min		0.14 ± 0.01	0.05 ± 0.01		0.07 ± 0.03	0.15 ± 0.01
13	9-C24:1	16.35 min	X	0.13 ± 0.01	0.04 ± 0.01	X	0.36 ± 0.02	1.08 ± 0.07
14	7-C24:1	16.45 min		0.04 ± 0.01	0.02 ± 0.01	X	0.08 ± 0.01	< 0.01
15	5-C24:1	16.63 min		0.15 ± 0.01	0.17 ± 0.02	X	0.15 ± 0.02	0.08 ± 0.01
16	C24:0	16.72 min	X	0.69 ± 0.01	0.41 ± 0.03	X	0.77 ± 0.03	0.61 ± 0.03
17*	C18:2-E^#^	16.95 min		0.23 ± 0.02	0.01 ± 0.01		0.18 ± 0.03	< 0.01
18*	C18:3-E^#^	17.06 min		0.59 ± 0.06	0.09 ± 0.03		1.17 ± 0.22	< 0.01
19	11^+^-C25:1	17.76 min	X	0.68 ± 0.03	3.69 ± 0.29	X	< 0.01	0.06 ± 0.03
20	9-C25:1	17.83 min	X	2.98 ± 0.11	3.50 ± 0.29	X	13.86 ± 0.40	6.44 ± 0.35
21	7-C25:1	17.93 min	X	2.83 ± 0.10	0.44 ± 0.06	X	27.37 ± 1.30	42.79 ± 1.39
22	5-C25:1	18.09 min	X	0.27 ± 0.01	0.85 ± 0.11	X	0.05 ± 0.02	0.95 ± 0.10
23	C25:0	18.20 min	X	24.77 ± 0.33	19.10 ± 0.77	X	18.69 ± 0.78	19.54 ± 1.32
24	11^+^-C26:1	19.22 min	X	0.19 ± 0.01	0.74 ± 0.03		0.00	0.00
25	9-C26:1	19.28 min	X	0.74 ± 0.02	0.55 ± 0.05	X	0.24 ± 0.03	0.06 ± 0.02
26	7-C26:1	19.40 min	X	0.43 ± 0.01	0.16 ± 0.02	X	0.50 ± 0.03	0.20 ± 0.01
27	5-C26:1	19.55 min		0.05 ± 0.01	0.32 ± 0.02		0.03 ± 0.01	0.07 ± 0.03
28	C26:0	19.65 min	X	0.47 ± 0.01	0.75 ± 0.02	X	0.43 ± 0.05	0.28 ± 0.03
29	C27:2	20.35 min		0.16 ± 0.01	0.13 ± 0.03		0.00	0.00
30	C27:2	20.44 min		0.40 ± 0.02	0.33 ± 0.05		0.00	0.00
31	11^+^-C27:1	20.64 min	X	1.91 ± 0.07	15.99 ± 0.66		0.00	0.00
32	9-C27:1	20.74 min	X	21.77 ± 0.43	13.68 ± 0.85	X	2.01 ± 0.16	0.14 ± 0.02
33	7-C27:1	20.85 min	X	10.95 ± 0.27	4.91 ± 0.48	X	6.20 ± 0.27	3.08 ± 0.17
34	5-C27:1	20.99 min	X	0.40 ± 0.02	7.72 ± 0.41	X	0.13 ± 0.02	0.40 ± 0.09
35	C27:0	21.07 min	X	4.45 ± 0.10	10.40 ± 0.38	X	4.40 ± 0.30	5.00 ± 0.44
36	11^+^-C28:1	22.09 min	X	0.02 ± 0.01	0.37 ± 0.02		0.00	0.11 ± 0.03
37	9-C28:1	22.15 min		0.43 ± 0.01	0.12 ± 0.01		0.12 ± 0.02	0.06 ± 0.02
38	7-C28:1	22.20 min		0.26 ± 0.01	0.28 ± 0.01	X	0.12 ± 0.01	0.04 ± 0.01
39	5-C28:1	22.32 min		< 0.01	0.12 ± 0.01		0.00	0.00
40	C28:0	22.40 min		0.33 ± 0.04	0.07 ± 0.01	X	0.31 ± 0.04	< 0.01
41	C29:2	23.16 min	X	1.36 ± 0.04	0.62 ± 0.04		0.00	0.00
42	C29:2	23.24 min	X	0.60 ± 0.02	0.34 ± 0.02		0.00	0.00
43	11^+^-C29:1	23.37 min	X	1.57 ± 0.05	4.20 ± 0.24		0.00	0.00
44	9-C29:1	23.45 min	X	3.05 ± 0.08	1.85 ± 0.10		0.59 ± 0.22	1.14 ± 0.13
45	7-C29:1	23.62 min	X	5.75 ± 0.16	5.12 ± 0.21	X	3.05 ± 0.26	1.16 ± 0.17
46	C29:0	23.82 min	X	0.71 ± 0.03	0.83 ± 0.08	X	1.71 ± 0.27	< 0.01
47	C31:2	25.96 min	X	1.26 ± 0.06	0.44 ± 0.09		0.00	0.00
48	7-C31:1	26.24 min	X	0.59 ± 0.02	0.06 ± 0.01	X	0.09 ± 0.01	0.05 ± 0.01
49	C31:0	26.38 min		0.17 ± 0.01	0.00	X	0.60 ± 0.10	0.03 ± 0.02

† Compound used to compute the discriminant analysis (DA)

^‡^ SE < 0.01 noted as 0.01

# Esters with unknown configuration of the alcohol

* Peaks found only in head extracts

11^+^ Double bond in position 11 or higher, to some extent a mixture of positional isomers of mono-alkenes resolved into one peak.

## Results

### Composition of female CHC profile

#### Osmia bicornis

**Fig 1:**
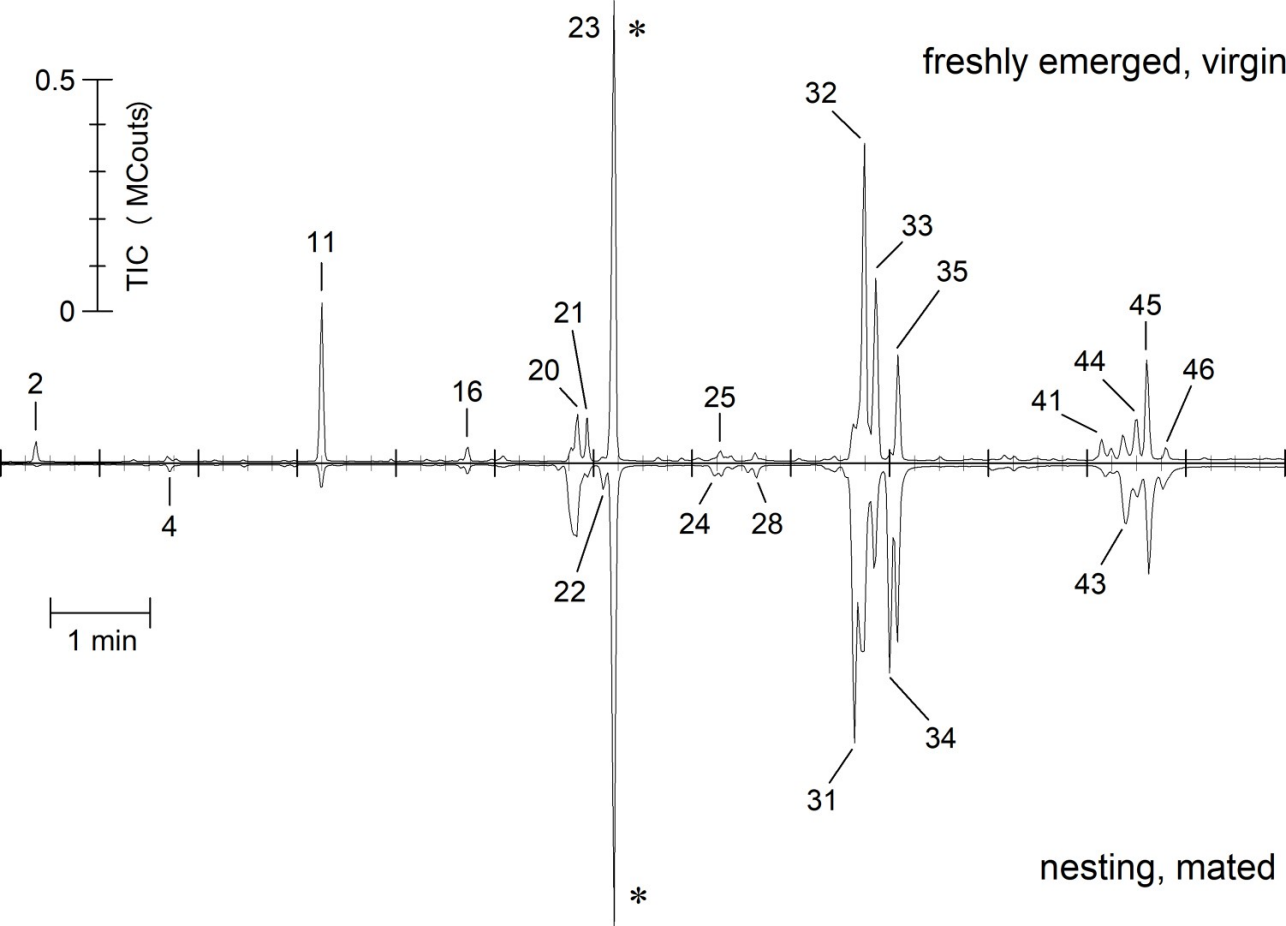
Contrasting juxtaposition of representative TIC-chromatograms of a freshly emerged, virgin (upper) and a nesting, mated (inverted) *O*. *bicornis* female in the retention time range of 12–25 min. Peak indicators refer to numbers in Table 1. Chromatograms are normalized to 1×10^6^ counts by the largest peak (indicated by an asterisk).

Pentane washings of freshly emerged (virgin) *O*. *bicornis* females (Fig 1) comprised a mix of series of n-alkanes (total TIC area for head washings: 39.07 ± 4.20%) in the range of 21 to 31 carbons chain length and their corresponding alkenes (total 55.82 ± 4.12%). The double bonds of n-alkenes were located in the positions 5- (1.22 ± 0.26%), 7- (20.92 ± 2.79%), 9- (29.16 ± 3.96%), 11- and higher (4.51 ± 0.83%). A tighter definition of the position of the double bonds in position 11 and higher was not possible due to the small amounts and the co-elution of this substances on the chromatographic system used, although in some cases indicators for position 12, 13, or 14 were found. Thus, n-alkenes with double bond position higher than 9 were summarised as 11^+^-alkenes. Alkadienes were found in small amounts (total 3.79 ± 0.84%) in chain lengths of 29 and 31 carbons, but the positions of the double bonds could not be identified. Carboxyl esters predominantly of hexadecenic acid with various not precisely identifiable alcohols were found throughout the chromatogram in trace amounts. Odd-numbered alkanes (37.91 ± 4.12%) and alkenes (52.89 ± 4.11%) predominated in general with pentacosane being the prominent alkane and heptacosenes the dominant alkenes, respectively (Fig 1, Table 1). Carboxyl esters as ethyl-octadecenoate were found in trace amounts only and could not be further specified.

**Fig 2:**
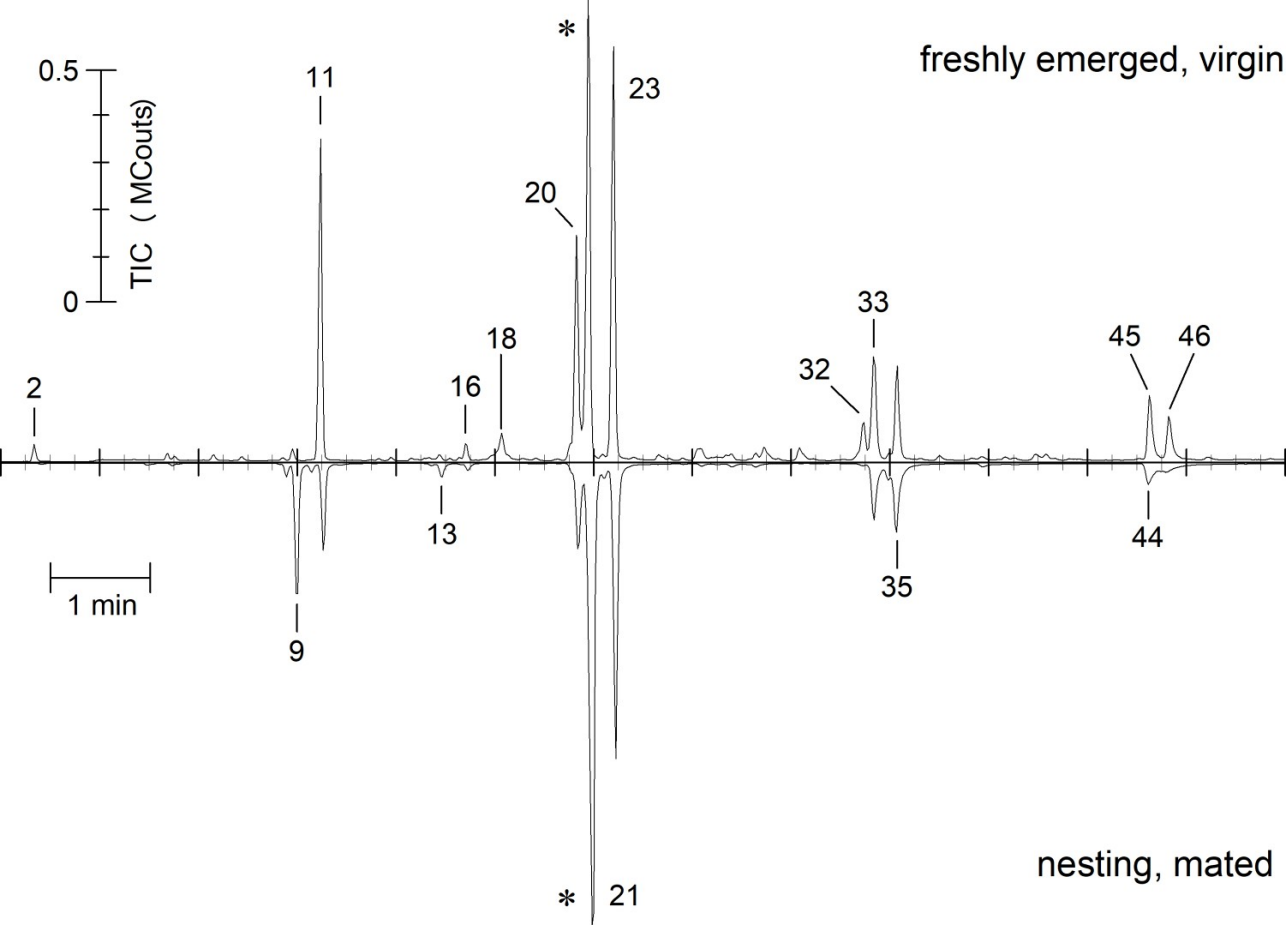
Contrasting juxtaposition of representative TIC-chromatograms of a freshly emerged, virgin (upper) and nesting, mated (inverted) *O*. *cornuta* female in the retention time range of 12–25 min. Peak indicators refer to numbers in Table 1. Chromatograms are normalized to 1×10^6^ counts by the largest peak (indicated by an asterisk).

**Fig 3:**
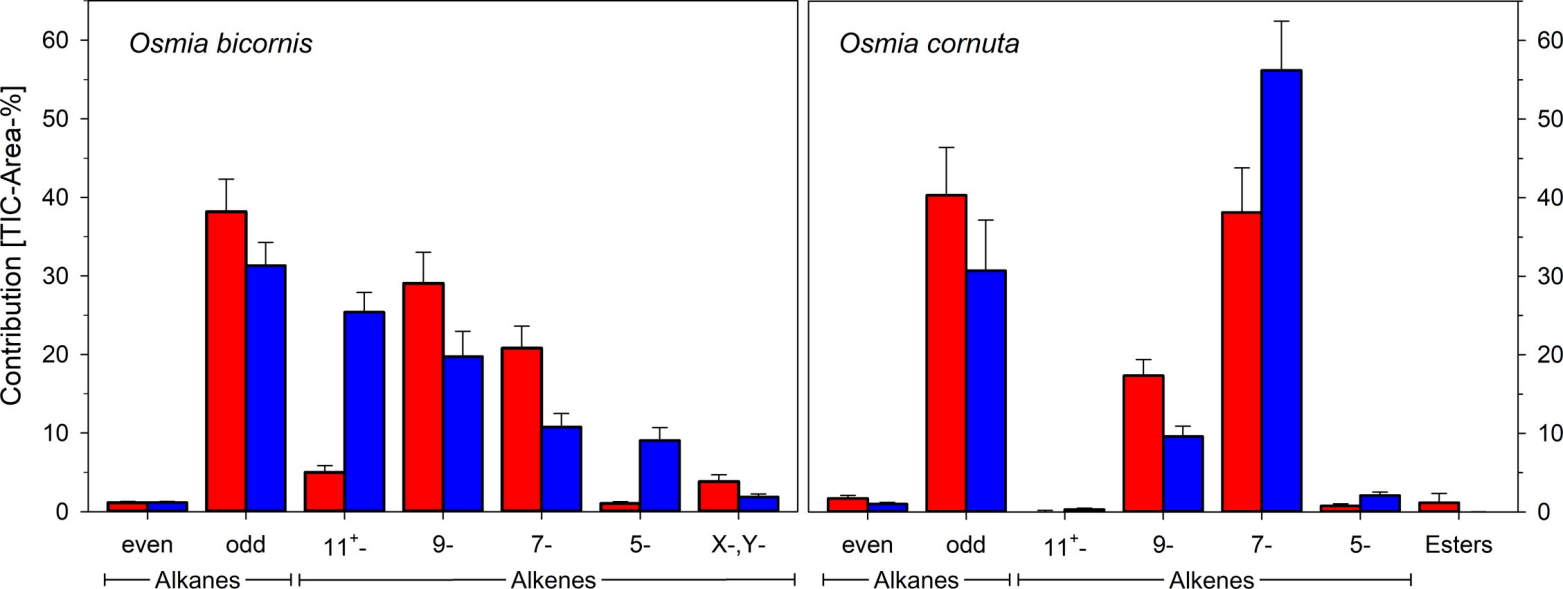
Composition of the CHC bouquet of *O*. *bicornis* and *O*. *cornuta* females. Red columns refer to freshly emerged (virgin), blue columns to nesting (mated) females, respectively. Whiskers indicate standard deviations; X-,Y-: alkadiens with unknown position of the double bonds.

***O*. *cornuta*** differed remarkably in its CHC composition from *O*. *bicornis* (Fig 2, Table 1). The content of alkanes (21 to 31 carbons chain length) is slightly higher (42.04 ± 6.35%) in freshly emerged, virgin females (Fig 3, right part). Alkenes contribute by 56.33 ± 6.80% to the CHC bouquet. Alkadiens and carboxyl esters were found in trace or small amounts, respectively. In contrast to *O*. *bicornis*, molecules having their double bond in position 7 dominate the alkene fraction.

### Transition of female CHC profile

#### Osmia bicornis

**Fig 4:**
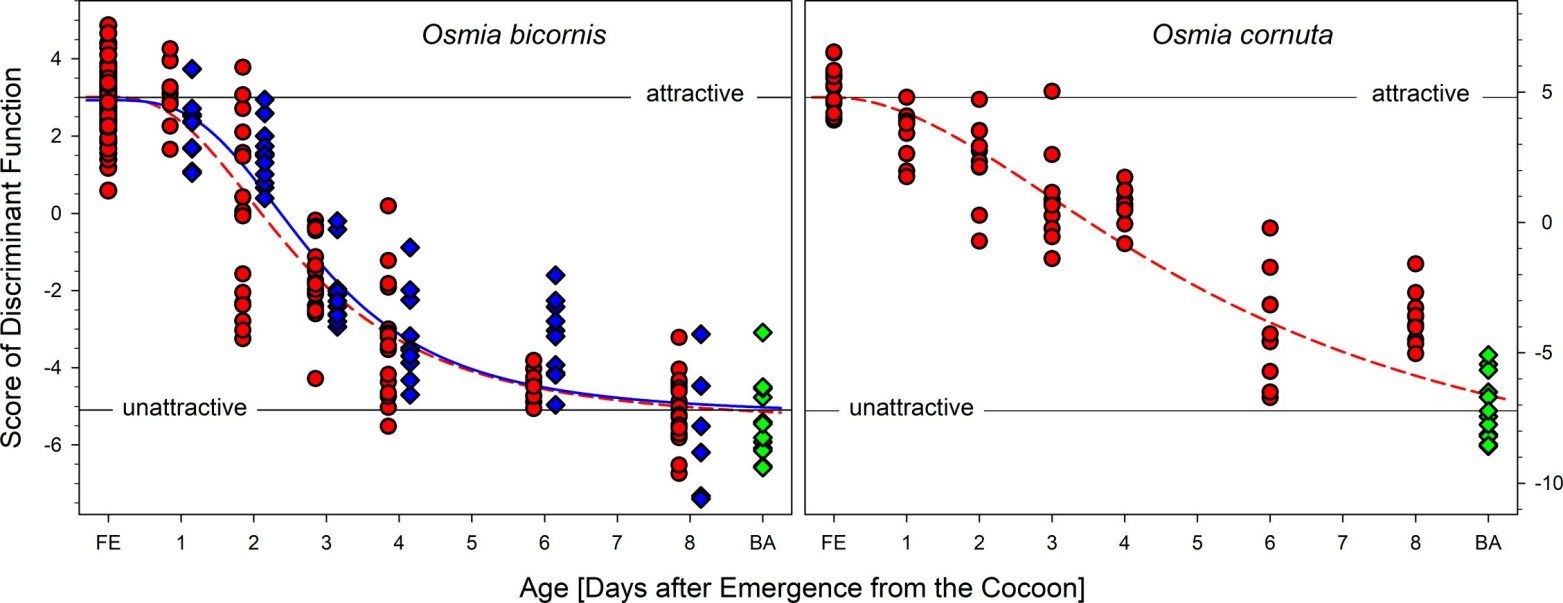
Shift of the discriminant function score as an estimator of attractiveness in *O*. *bicornis* (left panel) and *O*. *cornuta* (right panel) females on the course of the first days after emergence from the cocoon. Red circles: virgin females, blue diamonds: mated females, green diamonds: brood active (mated) females caught at nest blocks of the maintained populations; dashed lines indicate four parameter logistic regression of the discriminant score (*O*. *bicornis*: R^2^ = 0.88; *O*. *cornuta*: R^2^ = 0.84) for virgin females and solid line (R^2^ = 0.90) of mated females, respectively; FE: freshly emerged, BA: brood active.

**Fig 5:**
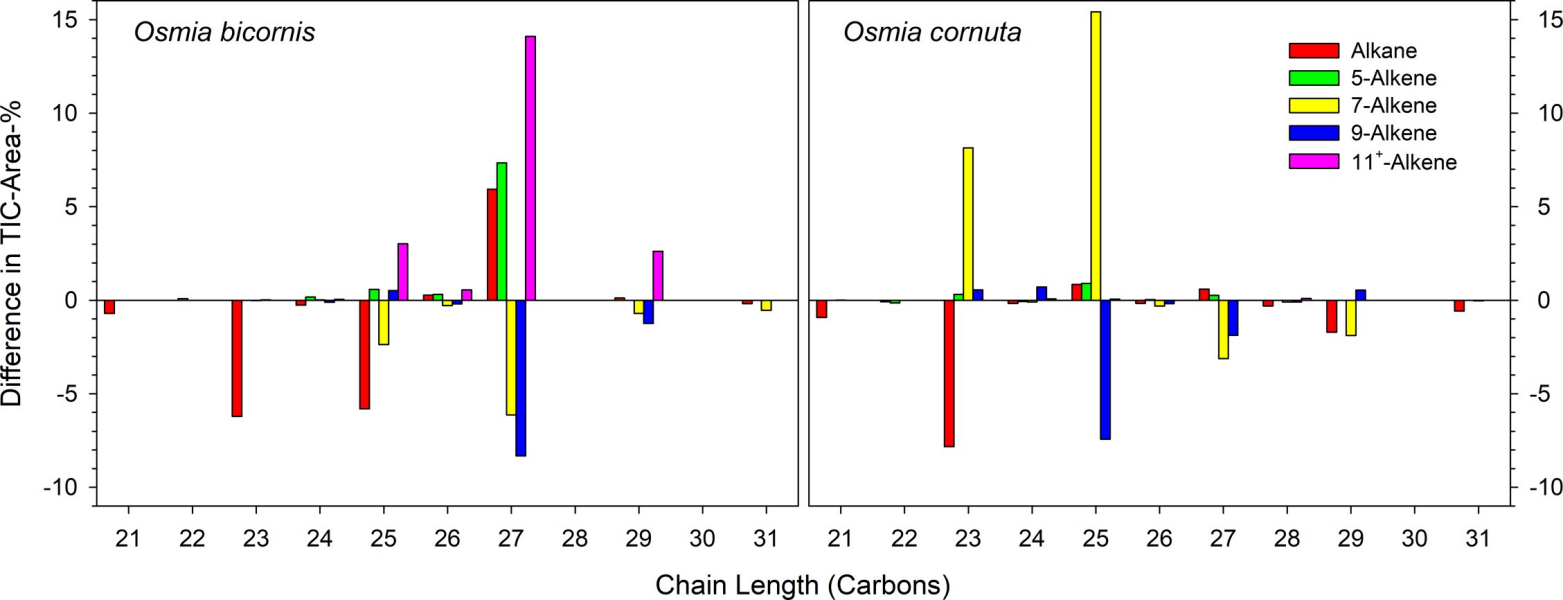
Differences in the proportion of main CHC components in nesting (mated) referred to freshly emerged (virgin) females (differences over all substances sum up to 0).

The composition of the hydrocarbons differed between freshly emerged (virgin) and brood active (mated) *O*. *bicornis* females (Fig 1, Table 1). The 30 components detectable in all individuals (total of 236 head samples of virgin and mated females in the age of 0 to 8 days or nesting) were reduced by PCA to 9 factors explaining 71.7% of the total variance. The scores of the canonical discriminant function modelled to distinguish freshly emerged (virgin) from brood active (mated) females (eigenvalue 20.235 accounting for 100% of the variance, λ = 0.047, χ^2^ = 288.760, P < 0.001) and calculated for all samples afterwards differed for females depending on their age but not on their mating status (GLM: virginity as fixed factor, age as a covariate; virginity: Wald-χ^2^_1, 161_ = 2.047, P = 0.153; age: Wald-χ^2^_1, 161_ = 353.596, P < 0.001). Thus, the volatile bouquet of females changed with age but was independent from an insemination (Fig 4). The changes in the hydrocarbon composition concern odd numbered alkanes and the composition of alkenes regarding the position of the double bond (Fig 3). The content of alkanes decreased to 32.47 ± 3.06% while alkenes increased to 65.56 ± 2.89%. The dominance of 9- and 7-alkenes declined in favour of alkenes with double bond in position 11 or higher as well as in position 5 (Fig 3). Most badly affected were alkenes with a chain length of 27 carbon atoms (Fig 5).

#### Osmia cornuta

The composition of the CHCs changed with age (Fig 2, Table 1). Altogether 30 components were detectable in all individuals (total of 78 head samples of virgin females in the age of 0 to 8 days and 13 head samples of nesting females) and reduced by PCA to 5 factors explaining 78.64% of the total variance. A canonical discriminant function distinguished freshly emerged (virgin) from nesting (mated) females (eigenvalue 40.193 accounting for 100% of the variance, λ = 0.024, χ^2^ = 98.534, P < 0.001). Females differed in the scores of the discriminant function depending on their age (Fig 4; GLM: age as a covariate; Wald-χ^2^_1, 78_ = 370.269, P < 0.001). The amount of odd numbered alkanes decreased in nesting females (total alkanes 31.75 ± 6.56%) while the prevalence of 7-alkenes had further increased (total alkenes 68.25 ± 1.82%; Fig 3). Most badly affected were CHCs with a chain length of 23 and 25 (Fig 5).

### Resemblance of CHC profiles from head and torso

#### Osmia bicornis

**Fig 6:**
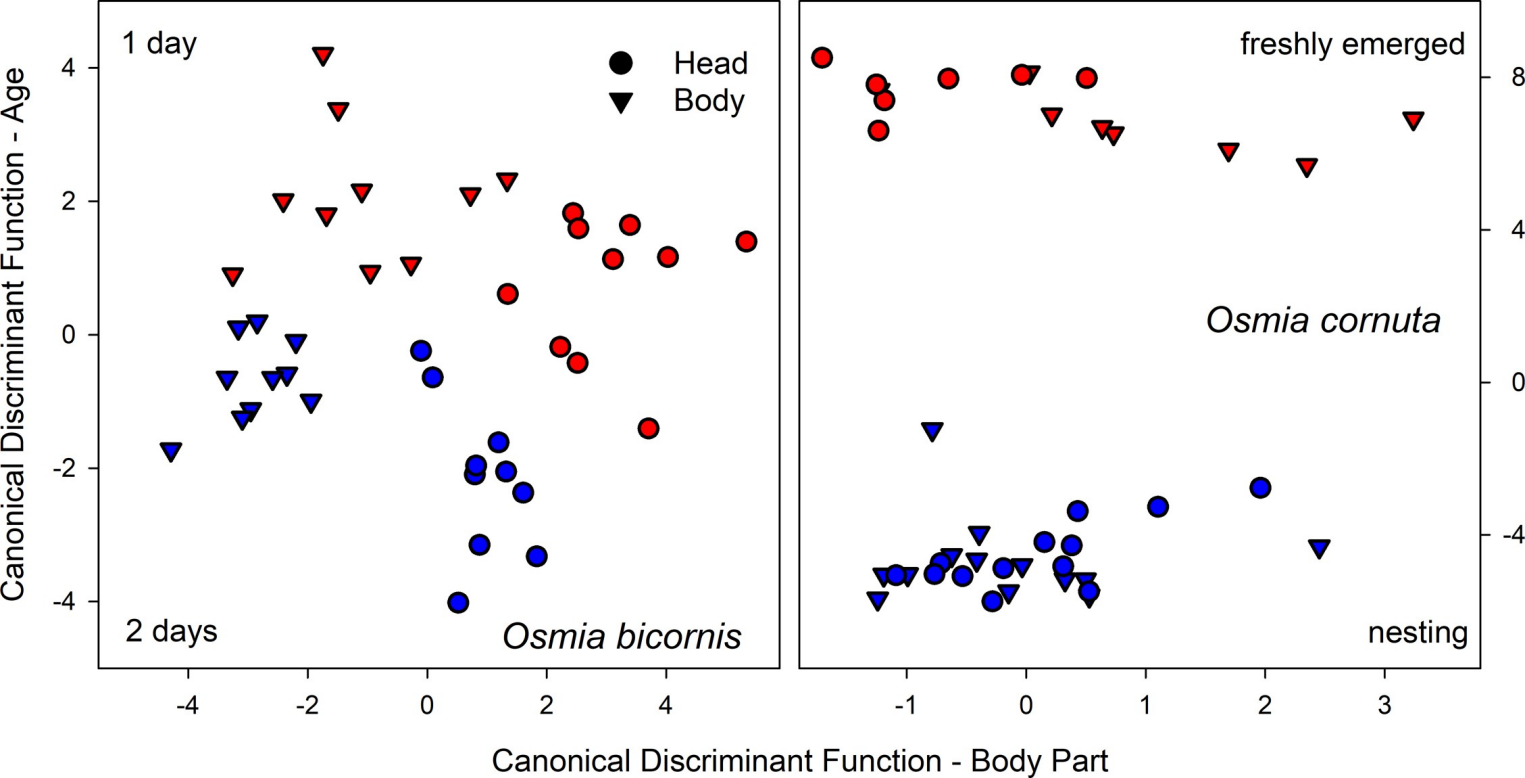
Cluster plot of the first two canonical discriminant functions for body parts and age cohorts. Left panel: *O*. *bicornis*, right panel *O*. *cornuta*. Circles and squares refer to heads and torsi, respectively. Red symbols indicate younger females, blue symbols mark older females (refer to the panel for the respective age).

The conformance of hydrocarbon composition from head and torso washings of one and the same individual was high. Nevertheless body parts were distinguishable (see below). The PCA of 33 hydrocarbons found both in head and torso washings extracted 7 factors explaining 80.47% of variability. By DA three canonical discriminant functions were found (eigenvalues 5.460, 2.773, 0.124) with the first two accounting for 98.5% of cumulated variance separating the four groups (body parts × age cohorts, Fig 6). Both body parts clustered co-directionally. Due to the differences in age, the conformity of head and torso extracts of individuals was separately analysed for the two age cohorts. In both groups, heads had lower Euclidean distances to their own torsi than to torsi of all other bees of the same age (age one day: SED_same_ = 7.276 ± 5.891, SED_alien_ = 14.131 ± 8.508, t = -2.477, df = 98, P = 0.015; age two days: SED_same_ = 7.074 ± 5.006, SED_alien_ = 11.065 ± 5.582, t = -2.165, df = 98, P = 0.033). Hence, both head and torso washes can be used to characterise changes in the volatile bouquet of females.

#### Osmia cornuta

The hydrocarbon composition from head and torso washings of one and the same individual was highly correlated although body parts were distinguishable by cluster analysis. The PCA of 30 hydrocarbons found both in head and torso washings extracted 5 factors explaining 79.70% of variability. The first two canonical discriminant functions (eigenvalue 38.077, 0.363) accounted for 99.6% of cumulated variance. The first discriminant function separated the age cohorts, while body parts were not clearly divorced (Fig 6). In both age groups, heads had lower Euclidean distances to their own torsi than to torsi of all other bees of the same age (Man-Whitney-U-test, freshly emerged: SED_same_ = 4.226 ± 2.756 {3.225, 1.854–9.720}, SED_alien_ = 11.674 ± 11.028 {6.417, 1.527–41.487}, U = 303.0, n = 63, P = 0.019; nesting: SED_same_ = 2.930 ± 3.290 {1.802, 0.145–11.096}, SED_alien_ = 7.411 ± 6.379 {5.776, 0.038–32.697}, U = 1469, n = 169, P = 0.004).

### The effect of mating on CHC transition in *Osmia bicornis*

The experience of a male post-copulatory display did not change the bouquet transition (GLM: manipulation of post-copulatory display (MpcD) as fixed factor, age as a covariate; MpcD: Wald-χ^2^_1, 121_ = 1.009, P = 0.315; age: Wald-χ^2^_1, 121_ = 27.283, P < 0.001). Inseminated females without post-copulatory display and virgin females that had received a post-copulatory display were classified according to their age. The scores from the canonical discriminant function did not differ from that of virgin or regularly mated females of the same age.

### Absence of male antiaphrodisiac on mated *O*. *bicornis* females

The assumed antiaphrodisiac 7-HDA-EE was not detectable in pentane washings of wings or bodies of freshly mated *O*. *bicornis* females (n = 10) sampled immediately after post-copulation. The diagnostic fragments of 236 m/z (C_16_H_28_O) and 237 m/z (C_16_H_29_O) could not even be tracked down in SIS mode of the MS in the corresponding retention time window. Appropriate, also the bodies of recently mated females one or two days after copulation (both samples n = 10) revealed no traces of 7-HAD-EE.

## Discussion

The CHC profiles of virgin females of the European mason bee species *O*. *bicornis* and *O*. *cornuta* constitute of series of acyclic aliphatics from C_21_ to C_31_ chain length. Odd-numbered alkanes and alkenes predominate. The most abundant positions of the double bond in alkenes were 9 and 7. Freshly emerged (virgin) and nesting (mated) females were clearly distinguishable both in *O*. *bicornis* and *O*. *cornuta* by a reduction of alkanes in favour of alkenes and a shift in the composition of the mono-alkene fraction regarding the position of the double bond. The transition was concluded within a week. The CHC profiles of heads and torsi changed co-directionally whereby tagmata of one individual were always more similar to each other than to other individuals. The general pattern of CHC assorts well to the profile found in the North American blue orchard bee, *O*. *lignaria* [21,22] and comes up to the general pattern of *Megachile rotundata* [9,21,22]. Odd numbered alkanes and alkenes (C_23_, C_25_, C_27_) dominated also the CHC profile of virgin *Amegilla dawsonia*, an anthophorinid ground-nesting, solitary bee [23]. Also in this species brood active females can be distinguished from virgin females by a drastically reduced content of alkanes in favour of alkenes [23]. Beside the characteristic changes in the CHC profile generally found in solitary bees, the bouquet of brood active females of ground nesting species becomes additionally enriched by substances produced by the Dufour’s gland that are used to line and impregnate the brood chambers [4,23,24].

**Fig 7:**
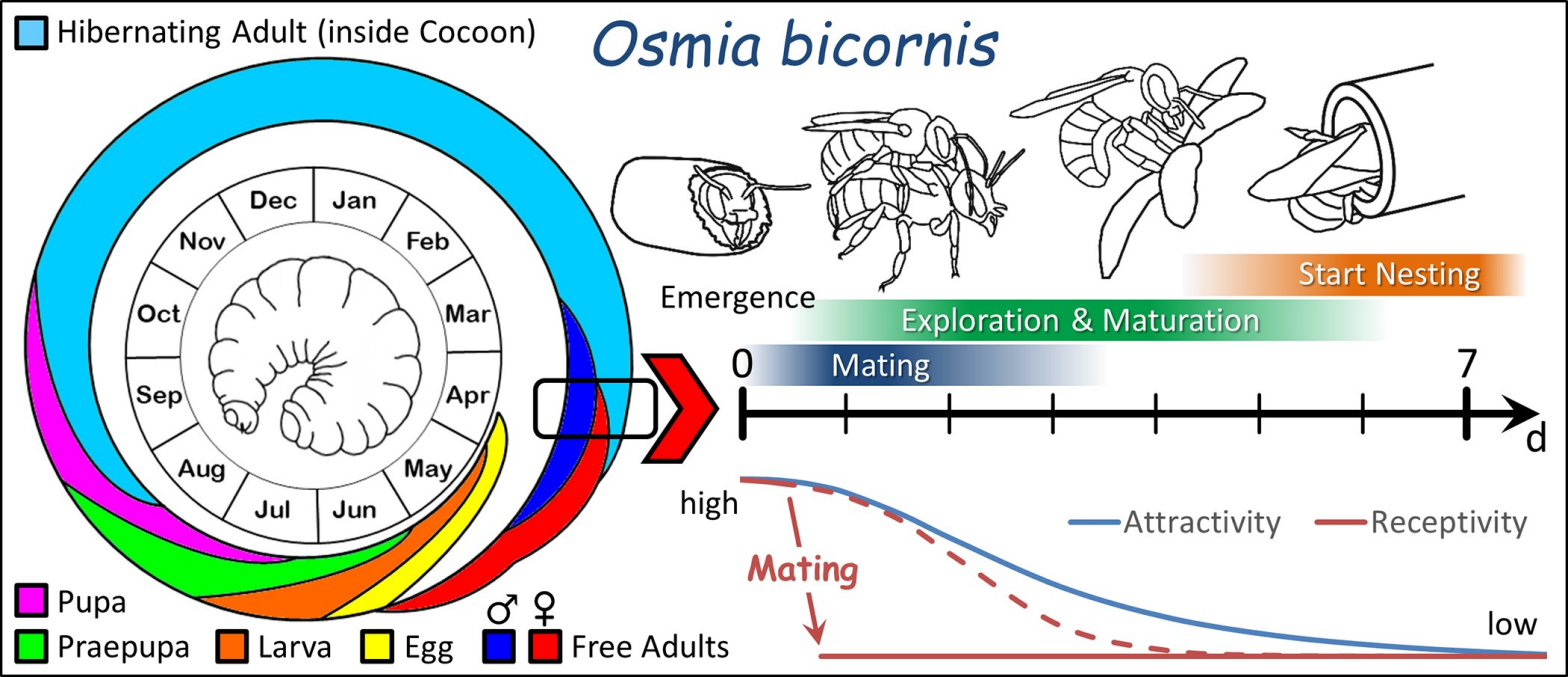
Life cycle of mason bees using the example of *O*. *bicornis*. Freshly emerged females possess a time window of a few days of receptivity (please note the inserted diagram) at the beginning of their life as free flying adults prior to the start of nesting activities.

The transition in the CHC profile of *O*. *bicornis* and *O*. *cornuta* was simply triggered by age and seems to be an autonomous ontogenetic process. There was no difference in the CHC profiles of virgin and mated *O*. *bicornis* females of the same age. Moreover, a closer analysis in this species demonstrated the independence of the bouquet from mating status in general or a storage of sperm in the spermatheca in particular as previously hypothesized [3,14]. Also the experience of a post-copulatory display by a male had no impact although this behaviour renders mated females immediately unreceptive [11]. However, the autonomous CHC profile transition is consistent to an age-dependent decrease in the proportion of virgin females that accepts an intromission when being courted by a male [11]. Both processes cause a short window of receptivity to mating after emergence from the cocoon in *O*. *bicornis* females (Fig 7) and probably in other *Osmia* species as well. Also a study addressing sexual attractivity in *M*. *rotundata* accentuated the importance of age for changes in the CHC profile [9].

An ongoing sexual harassment by males interferes with mated female’s interest to establish a nest and provision brood cells unimpededly. Therefore, it is in the interest of both mating partners to protect the inseminated female from further chasings by males searching for copulation opportunities [25]. Consistent to this theoretical background, the induction of unreceptivity along with reduced attractiveness due to an antiaphrodisiac was found in several insect species [26,27,28]. Males of both studied mason bee species possess a sternal gland with hairs forming a brush that is predestined to paint the pheromone on surfaces as the male’s hind legs to impregnate a mated female (personal observations). Unexpectedly, the proposed antiaphrodisiac 7-HDA-EE [3] was not detectable even in traces on mated *O*. *bicornis* females after a copulation. The described occurrence of the substance immediately after a copulation on wings [3,14] could not be reproduced in this study. Perhaps the trove originated from a contamination due to close body contact during prae-copula and intromission and not from brushing the substance on the wings within the post-copula [4]. The deficiency of 7-HDA-EE on mated females’ reopens the question of the function of the sternal gland secrete that comes up to all expectations of a pheromone. This result can probably be extended to *O*. *cornuta* and other mason bees with a post-copulatory display as *O*. *lignaria* and *O*. *cornifrons* [11]. Males of these species possess also a sternal gland (personal observations) although the chemical identity of the secretions is up to date unknown.

How do the exceptional findings of an autonomous transition towards an unattractive CHC profile in young females regardless of an insemination and the lack of an antiaphrodisiac despite high male harassment fit into the biology of the studied mason bees *O*. *bicornis* and *O*. *cornuta*? Males of both species intensively compete for virgin females due to the distinct male biased sex ratio and monandry of females [29,30,31,32]. For nest construction, females of both species use pre-existing cavities that are usually scattered over the habitat and visit a wide range of forage plants for pollen and nectar. Neither nesting sites as sources of virgin (emerging) females nor food resources can be monopolised by males. Instead males engage in a race-like scramble competition for matings by searching nesting sites and patrolling flowers as well [30]. The accumulated demand of males for copulations widely dispenses females from active mate finding activities. The expenditure to locate a mate is defrayed by males alone. Young females leave the maternal nest after emerging from their cocoon and stray through the habitat for the first few days (personal observations) to feed on flowers and to search for a suitable cavity to construct their first own nest (Fig 7). During this time, they are likely to be detected by searching males. It can be hypothesised that males increase the rate of encountering virgin females by marking the flowers or plants they patrol as it is known from bumble bees ([33] and references therein). The secretions of the sternal gland could perfectly serve as a marking pheromone of *Osmia* males to announce their presence. Applied at plants or flowers, such pheromone marks could guide virgin females to locations where they are likely to become detected by a male even at low population densities. The combination of intensively searching males with a male marking pheromone could ensure the insemination of virgin females within the short time window of receptivity by such a certainty that the moderate CHC alteration does not need a specific trigger. The bouquet transition can be simply coupled to ontogenetic, probably physiological processes as e.g. the juvenile hormone driven maturation of the ovary. Despite the moderate rate of CHC transitions, females become distinguishable by conspecific males already after one day [7]. Thus, after the pre-nesting period of reproductive maturation females have rendered their initially attractive CHC profile unattractive and can start nesting also without the protection against male sexual harassment by an antiaphrodisiac.
